# Extraction, Segmentation, and 3D Reconstruction of Wire Harnesses from Point Clouds for Robot Motion Planning

**DOI:** 10.3390/s25247542

**Published:** 2025-12-11

**Authors:** Saki Komoriya, Hiroshi Masuda

**Affiliations:** Department of Mechanical Engineering and Intelligent Systems, Graduate School of Informatics and Engineering, The University of Electro-Communications, Tokyo 182-8585, Japan; saki.komoriya@uec.ac.jp

**Keywords:** point cloud, digital twin, wire harness, branch detection, generalized cylinder, motion planning, virtual environment, point cloud processing

## Abstract

Accurate collision detection in off-line robot simulation is essential for ensuring safety in modern manufacturing. However, current simulation environments often neglect flexible components such as wire harnesses, which are attached to articulated robots with irregular slack to accommodate motion. Because these components are rarely modeled in CAD, the absence of accurate 3D harness models leads to discrepancies between simulated and actual robot behavior, which sometimes result in physical interference or damage. This paper addresses this limitation by introducing a fully automated framework for extracting, segmenting, and reconstructing 3D wire-harness models directly from dense, partially occluded point clouds captured by terrestrial laser scanners. The key contribution lies in a motion-aware segmentation strategy that classifies harnesses into static and dynamic parts based on their physical attachment to robot links, enabling realistic motion simulation. To reconstruct complex geometries from incomplete data, we further propose a dual reconstruction scheme: an OBB-tree-based method for robust centerline recovery of unbranched cables and a Reeb-graph-based method for preserving topological consistency in branched structures. The experimental results on multiple industrial robots demonstrate that the proposed approach can generate high-fidelity 3D harness models suitable for collision detection and digital-twin simulation, even under severe data occlusions. These findings close a long-standing gap between geometric sensing and physics-based robot simulation in real factory environments.

## 1. Introduction

In manufacturing plants, many articulated robots are installed, and they must operate without colliding with each other. In general, manual teaching, in which operators directly manipulate the robots, requires considerable time and effort. Therefore, off-line robot teaching, performed prior to on-site teaching, is an effective approach. Off-line teaching commonly utilizes CAD models of articulated robots.

In factory environments, robots are equipped with various wire harnesses for power and signal transmission. However, accurate 3D models of wire harnesses are rarely created. Because the harnesses are typically installed with slack to accommodate the robot’s movements, it is difficult to model them precisely during the design phase. Moreover, their mounting positions are often determined empirically by experienced workers on the factory floor, making their exact configurations hard to predict in advance. The clamping positions and attachment methods for wire harnesses vary depending on the robot’s geometry and the type of end-effector, making it labor-intensive to create detailed CAD models for each robot. If wire harnesses are not modeled, however, their motion cannot be simulated during off-line teaching, resulting in discrepancies between simulated and actual robot behavior. Unintended harness motion may cause interference between robots or even lead to mechanical damage.

In practice, wire harnesses present several inherent challenges for geometric reconstruction. Large portions of the harness surface are typically missing in point clouds because the backside is occluded by robot links, clamps, or surrounding devices. Furthermore, the harness shape is not rigid: slack produces irregular sagging and deformation that vary across robot poses. Consequently, the resulting point clouds are highly incomplete, anisotropic, and non-watertight, making them difficult to process using conventional 3D reconstruction techniques.

**Figure 1:**
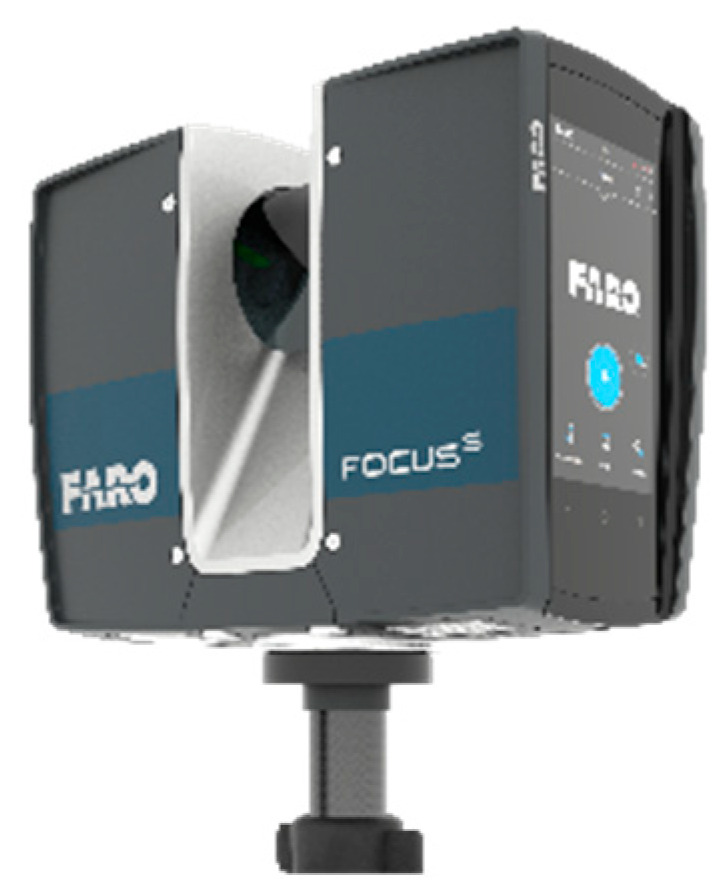
Terrestrial laser scanner.

In recent years, terrestrial laser scanners have enabled the acquisition of dense point clouds of manufacturing plants. Figure 1 shows an example of a terrestrial laser scanner. These scanners can capture the 3D geometry of factory equipment as point clouds consisting of millions to billions of points. By extracting wire harnesses from these data and converting them into 3D mesh models for integration with robot CAD models, more accurate interference analysis can be achieved. This process requires isolating movable components, such as wire harnesses, from the factory’s point cloud data and replacing them with corresponding 3D models.

Wire harnesses can be broadly categorized into two types: those fixed to the robot body and those that change their relative position as the robot moves. The latter cannot simply be attached to the robot model, as their motion is independent of the robot’s rigid links. In such cases, each harness must be modeled separately, and dynamic motion simulations need to be performed to reproduce its behavior accurately. Even when the harness is directly attached to the robot, it is still important to identify the appropriate link for attachment and to segment the harness point cloud for each link. These segmented point clouds then serve as the basis for reconstructing explicit 3D geometric models of the harnesses, which are required for downstream analysis such as interference checking and motion simulation.

Moreover, the reconstructed 3D models are intended to be used in physics-based motion simulation, where the dynamic harness segments are treated as flexible bodies constrained at clamp locations. Although detailed simulation modeling is beyond the scope of this paper, the reconstructed centerlines and radii provide the geometric foundation required for applying such constraints within common physics engines.

Many researchers have investigated 3D model reconstruction from point cloud data [1,2,3,4,5]. Poisson surface reconstruction [6], for example, is widely used to generate smooth 3D surfaces from dense point clouds. However, it often fails to produce accurate results when multiple objects are in close proximity or when the data contain severe occlusions. Since it is impractical to capture complete point clouds of numerous wire harnesses in large-scale factory environments, such methods are not well suited for harness modeling.

In the field of industrial facility modeling, numerous studies have focused on detecting and reconstructing piping structures [7,8,9]. For instance, Midorikawa et al. [10] modeled pipes by extracting generalized cylinders from point clouds. However, these methods depend on identifying circular cross-sections to define the pipe geometry, which limits their applicability to wire harnesses. Point clouds of wire harnesses often contain large missing portions due to occlusions, and their centerlines must be estimated as highly flexible freeform curves, which poses challenges distinct from those of rigid, static piping.

Many studies on mesh processing have addressed skeletonization, which aims to extract the centerline (skeleton) from arbitrary 3D shapes. Laplacian-based contraction [11,12], a well-known approach, iteratively shrinks the shape toward its medial axis, thereby effectively capturing its topology. However, this technique is primarily designed for closed, watertight meshes and is therefore unsuitable for unclosed, partial point-cloud surfaces obtained from terrestrial laser scanners. Because the entire backside of a wire harness is often missing, the Laplacian averaging process tends to cause the estimated centerline to deviate significantly from the true geometry. Hence, a different and more robust approach is required to estimate the centerline from such incomplete data.

Recent studies have also addressed the geometric reconstruction of wire-like structures such as railway catenaries and high-voltage transmission lines [13,14,15]. These wires are typically characterized by small diameters and are constrained only at their endpoints, allowing their trajectories to be represented by analytic geometric curves such as catenaries. However, robot wire harnesses generally have larger diameters and follow more complex routing paths to accommodate the robot’s motion.

Although many reconstruction and skeletonization techniques have been proposed, they generally assume watertight surfaces, complete cross-sections, or uniformly sampled points. TLS-derived wire-harness point clouds violate all of these assumptions: they contain severe occlusions, missing backside surfaces, irregular sampling density, and sometimes multi-branch structures. Existing methods thus fail to recover stable centerlines or correct topology under real factory conditions, leaving a clear technical gap that must be addressed.

To overcome these limitations, this paper proposes a comprehensive method to extract, segment, and reconstruct wire harnesses from incomplete point clouds of articulated robots. The main contributions of this study are summarized as follows:

- Integrated Pipeline: A fully automated framework for processing large-scale factory point clouds to generate collision-ready mesh models of wire harnesses.
- Motion-Based Segmentation: A novel classification method that distinguishes between static harnesses (fixed to links) and dynamic harnesses (moving independently), enabling more realistic kinematic simulations.
- Robust Reconstruction Strategy: A hybrid centerline estimation approach combining OBB-trees and Reeb graphs, specifically designed to handle non-watertight surfaces, backside occlusions, and missing data.
- Real-World Validation: Experimental evaluation on articulated robots installed in an operational automotive assembly plant, demonstrating effectiveness in terms of reconstruction accuracy and computation time.

The following sections provide an overview of the proposed method. Section 3 presents the extraction of wire harness point clouds from the overall robot point cloud. Section 4 describes the segmentation of the wire harness point clouds into parts that move relative to the robot links and parts that are fixed to them. Section 5 introduces the shape reconstruction approach for unbranched wire harnesses. Section 6 explains the centerline estimation and 3D model generation methods for branched wire harnesses. Finally, Section 7 presents the experimental results and conclusions.

## 2. Overview

**Figure 2:**
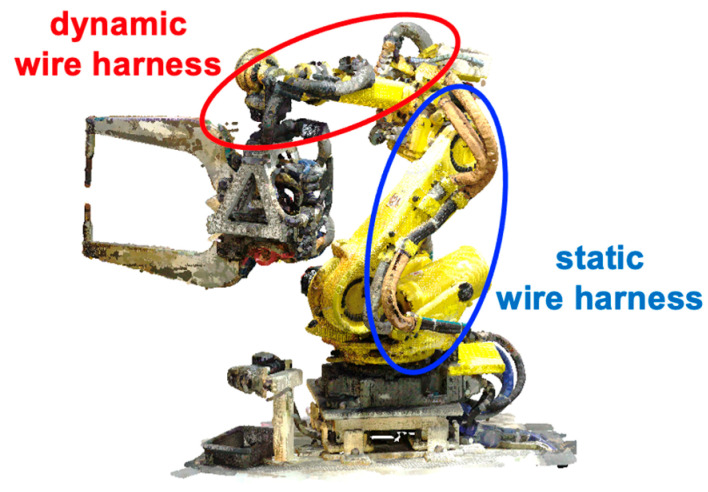
Two types of wire harnesses.

Figure 2 shows point clouds of an articulated robot captured using a terrestrial laser scanner. The data were acquired from multiple scanning locations. In this paper, we describe our method using the point clouds of this robot, which consists of 10 links performing rotational or translational motion. A wire harness runs along the robot structure, extending from the base to the end effector.

**Figure 3:**
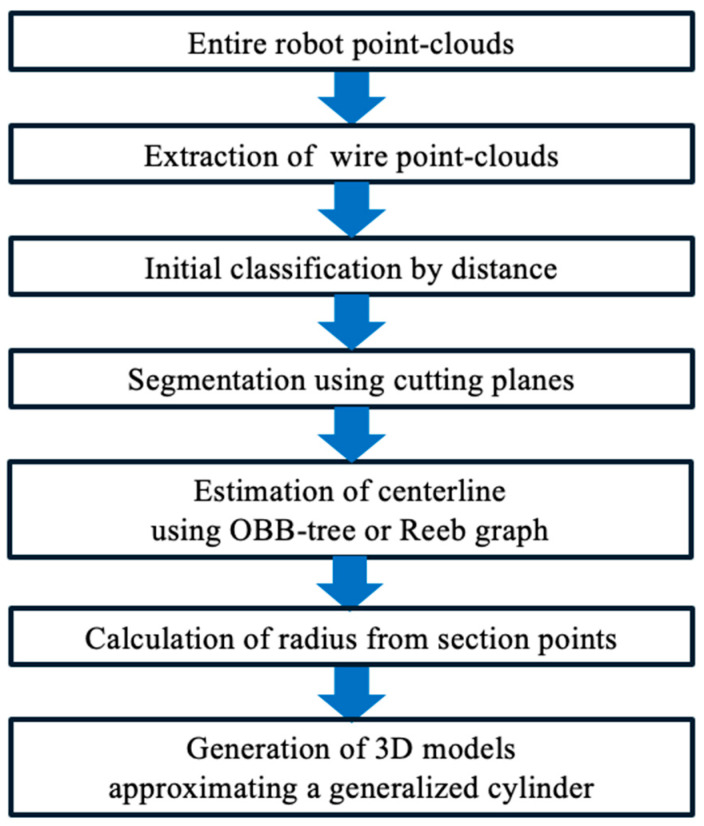
Outline of the method.

An outline of the proposed method is shown in Figure 3, which provides a flowchart of the overall processing pipeline. First, wire harness point clouds are extracted from the robot point clouds. Because the harness is attached along the robot links, directly isolating it is difficult. Fortunately, CAD models of most industrial robots are available from robot manufacturers. Therefore, we first align the CAD model of the articulated robot to the point-cloud data and remove the points corresponding to the robot links. The remaining points serve as candidates for wire-harness points, from which we detect the actual wire harness point clouds.

Wire harnesses include both static portions that are firmly fixed to each link and dynamic portions that move relative to the link due to slack, as illustrated in Figure 2. In our method, harnesses are classified into these two types. Each type is then detected and segmented using cutting planes perpendicular to the axis of each robot link.

The extracted harness point clouds often contain substantial missing regions. The laser beam cannot reach the backside of the harness when it is tightly attached to a link, covered by clamps, or partially occluded by surrounding objects. As a result, it is difficult to detect a continuous centerline directly from such incomplete data. To address this, we estimate the centerline by obtaining representative points from oriented bounding boxes (OBBs) and connecting them to form a continuous curve. For harnesses with branches, we employ a Reeb graph [16] to determine the centerline topology. The estimated centerlines are then approximated using B-spline curves to smoothly interpolate missing segments.

The wire harness is modeled as a generalized cylinder. The 3D model is generated by sweeping circles of varying radius, computed from cross-sectional points along the centerline. The effectiveness of the proposed method is demonstrated using point clouds of articulated robots installed in an automotive manufacturing environment.

## 3. Extraction of Candidate Points for Wire Harnesses

**Figure 4:**
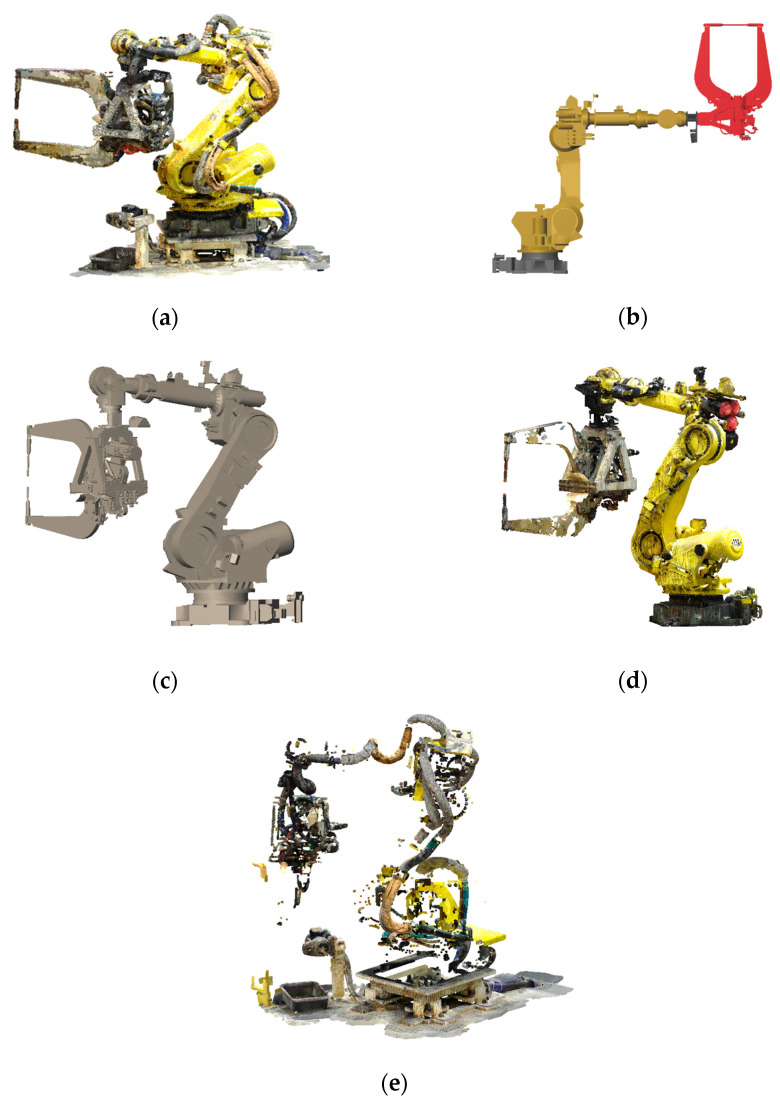
Extraction of candidate points for wire harnesses: (**a**) Point cloud of an articulated robot; (**b**) Initial CAD models in the assembled configuration; (**c**) CAD models fitted to the point cloud; (**d**) Points corresponding to the robot links; (**e**) Remaining points after removing the robot links.

This section describes the extraction of candidate point clouds for wire harnesses. Figure 4a shows the point cloud of an articulated robot with wire harnesses attached along its links. To isolate the harness point clouds, we first detect and remove the points corresponding to the robot links from the original point cloud. Figure 4e shows the resulting point cloud after removing the link regions. The remaining points constitute the candidate points from which the wire harnesses will be identified.

CAD models of articulated robots used in factory environments are typically provided by robot manufacturers mainly for off-line teaching. In this study, we assume that the movable parts of the robot are available as independent 3D models and that a complete assembled CAD model of the robot is also provided.

The proposed method uses these CAD models to separate the point clouds associated with individual wire harnesses. We first align the CAD models of the robot links to the measured point cloud using the method of Kawasaki et al. [17] and then remove the corresponding link regions from the point clouds. This fitting method enables precise alignment of each link model with the measured data.

In Kawasaki’s method, the CAD model of each link is sequentially fitted to the point cloud, starting from the base link and proceeding toward the end effector by exploiting the kinematic relationships between connected links. In this procedure, a constrained ICP formulation is employed in which the rotational axis of each CAD link is required to coincide with the axis of the previously aligned link. This constraint allows the link models to be fitted sequentially and consistently to the measured point cloud. Figure 4b shows the initial CAD models of the robot links, and Figure 4c illustrates the fitted models after applying this procedure.

Once the CAD models are aligned, the point clouds corresponding to the robot links can be extracted by selecting the points within a specified distance from the fitted link surfaces, as shown in Figure 4d. In this study, we used a neighbor distance of 3 cm. Candidate wire-harness points are then obtained by removing these link points from the original point cloud, as shown in Figure 4e. The remaining points correspond to those not represented by the CAD models and include not only wire harnesses but also other components such as clamps, ground-mounted devices, and stands.

## 4. Segmentation to Static and Dynamic Wire Harnesses

After extracting candidate point clouds for the wire harnesses, the points are categorized into static wire harnesses, which are fixed to the robot links, and dynamic wire harnesses, which move relative to the links due to slack.

### 4.1. Extraction of Neighbor Points of Robot Links

**Figure 5:**
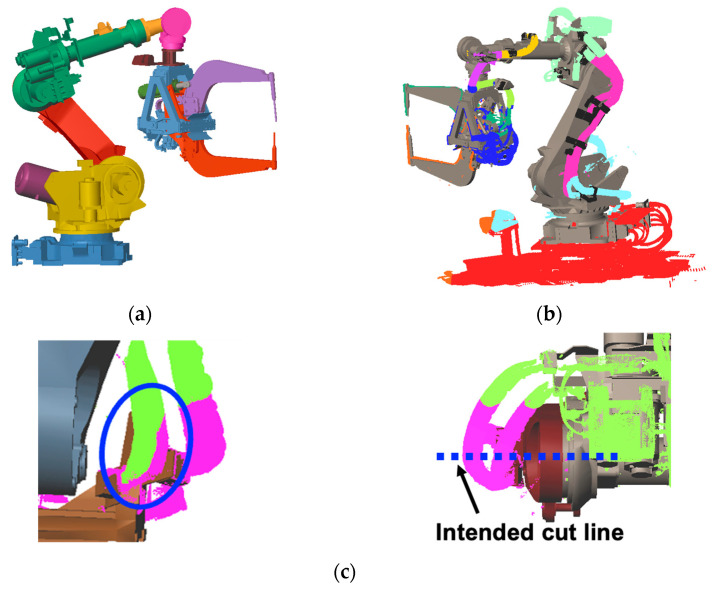
Initial segmentation using the closest robot links: (**a**) Aligned CAD models of robot links; (**b**) Wire harnesses segmented by the closest robot links; (**c**) Inadequate segmentation.

Candidate wire-harness points are extracted based on their distance to the robot links. Figure 5a shows the aligned CAD model of the robot links; each indicated in a different color. For each point, the nearest robot link is identified. Points whose distance exceeds a predefined threshold are regarded as unrelated to the wire harness and removed.

Figure 5b illustrates the resulting segmentation, where points are grouped by their nearest robot link and removed points are shown in red. This produces an initial, coarse classification of candidate points for each link.

### 4.2. Segmentation of Static Wire Harnesses

Static wire harnesses are rigidly fixed to the robot links and therefore move together with them. However, the coarse segmentation based solely on the nearest link may incorrectly assign some points, as shown in Figure 5c. To correct this, we refine the boundaries so that the wire harness is segmented precisely at the joint between two connected links.

**Figure 6:**
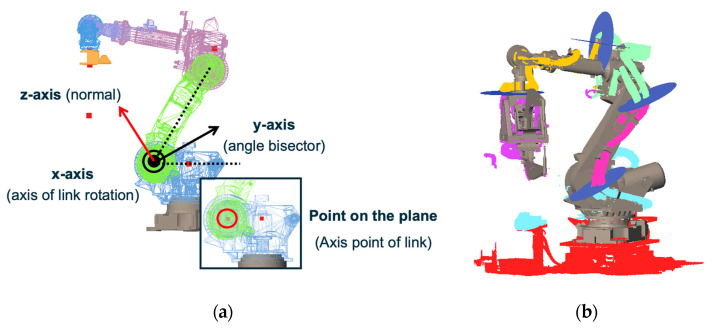
Cutting-plane definition and segmentation refinement for static wire harnesses: (**a**) Definition of the cutting-plane axes; (**b**) Refined segmentation using cutting planes placed along the rotation axes.

To segment the wire harness for each link, a cutting plane is defined at the rotation axis between the two links. As shown in Figure 6a, the plane is defined by the *x*-axis and *y*-axis, where the *x*-axis corresponds to the rotation axis of the joint, and the *y*-axis is the bisector of the angle formed by the two links. The *z*-axis becomes the normal vector of the cutting plane. The position of each cutting plane is computed from the intersection of the rotation axis and the wire-harness point cloud.

Figure 6b show the cutting planes (in blue) together with the refined segmentation results, which are color-coded for each links. By slicing the initial segmentation with these planes, appropriate segment boundaries are obtained.

### 4.3. Segmentation of Dynamic Wire Harnesses

Dynamic wire harnesses are those that are not rigidly fixed to the robot links and may move independently due to slack. In this study, a wire-harness segment is regarded as dynamic when the candidate points obtained from the coarse segmentation in Figure 5b exhibit a deflection from the nearest robot link that exceeds a predefined threshold. Such displacement indicates that the harness is not constrained by the link and can move during robot operation.

**Figure 7:**
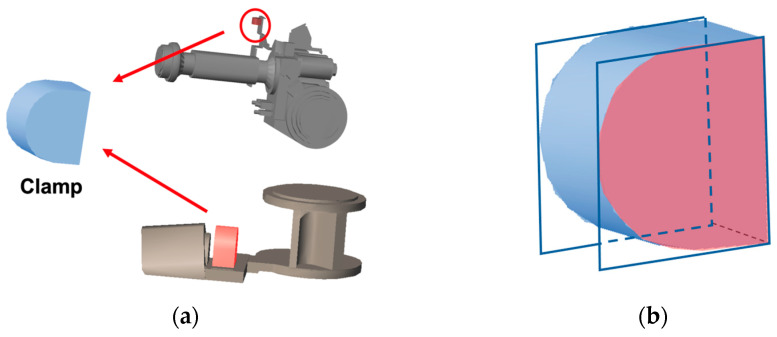
Boundary refinement for dynamic wire harnesses: (**a**) Clamp positions and 3D CAD model; (**b**) Cutting planes defined at the clamp surfaces.

Dynamic wire harnesses are secured to the robot at specific clamp positions. Figure 7a shows the CAD model of a clamp. We assume that the clamp geometry is included in the CAD models of the robot links. Each clamp has an upper and a lower mounting surface, and for dynamic harnesses, these two surfaces are treated as cutting planes for refining the segmentation boundaries. Figure 7b illustrates the cutting planes defined at the clamp surfaces. By slicing the candidate harness points using these planes, we obtain accurate boundaries for dynamic wire harness segments.

After applying the clamp-based cutting planes, one of the resulting segments may lie closely along a robot link and show no significant slack. When this occurs, the segment is treated as a static wire harness. Its boundary is then further refined using the cutting planes defined at the rotation axes of the robot links, as described in Section 4.2.

We note that clamp models are sometimes omitted from the CAD data provided for industrial robots. In such cases, our implementation requires the user to manually specify the position and orientation of each clamp on the CAD model.

## 5. Generation of 3D Mesh Models for Dynamic Wire Harnesses

In this section, we describe the process of generating a 3D model for each wire harness from the segmented point clouds. Static wire harnesses are rigidly attached to robot links and move together with them. Therefore, their geometry can be used directly as point clouds for collision checking. In contrast, dynamic wire harnesses move independently of the robot links because they contain slack. To simulate their motion and evaluate potential interference accurately, it is necessary to construct a mesh model that can be used in dynamic analysis. For this reason, we generate a mesh model from the point clouds of each dynamic wire harness.

A wire harness can be approximated as a generalized cylinder, defined by an arbitrary centerline and a variable radius. Thus, both the centerline and the radius must be estimated from the point cloud. This section describes the centerline estimation method for wire harnesses without branches.

### 5.1. Estimation of the Centerline of Wire Harness

**Figure 8:**
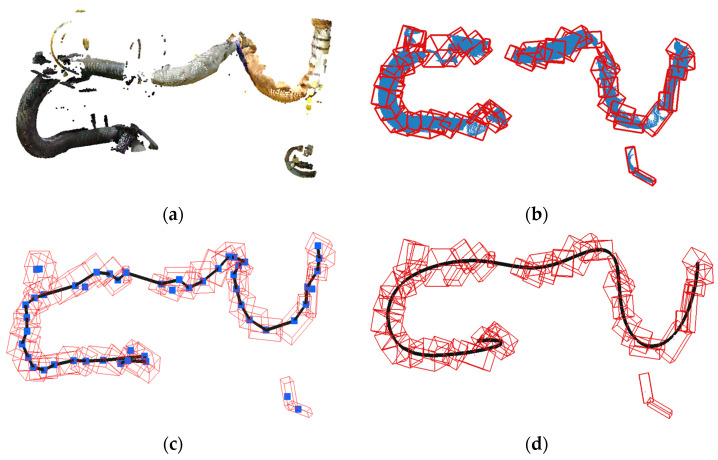
Estimation of centerline using OBB-tree: (**a**) Point-clouds of dynamic wire; (**b**) Generated OBB-tree; (**c**) Detected centerlines; (**d**) Smoothed centerline.

Figure 8a shows the point cloud of a dynamic wire harness. Portions of the wire harness are often missing on the backside, around clamps, or in occluded regions, making direct centerline estimation unreliable.

Before estimating the centerline, residual points that are not part of the harness are removed to ensure robustness. The input point cloud P is first converted into a graph by connecting spatially neighboring points, following Step 1 of Algorithm 1. This graph is then decomposed into independent connected components, and any isolated component containing fewer points than a predefined threshold is treated as noise and discarded. This removes the residual points visible ensuring that they do not affect the subsequent OBB-tree construction.

To obtain a stable centerline from the filtered point cloud, the data must be subdivided into small regions from which representative points can be extracted reliably. This subdivision is performed using an OBB-tree constructed exactly as described in Algorithm 1. Each subset S is enclosed by an oriented bounding box (OBB) whose orientation is determined from the eigenvectors of its covariance matrix. If $|S|<N_{min}$ or the OBB length falls below $L_{max}$, the OBB center is recorded; otherwise, the subset is recursively split along a plane perpendicular to the principal direction $e_{1}$. Figure 8b illustrates the resulting OBBs (red boxes) and the remaining wire-harness points (blue points).

The centers of all terminal OBBs form the set Centers, which serves as the input for polyline extraction. To estimate the centerline, these centers are connected using a growing procedure consistent with Step 3 of Algorithm 1. An arbitrary center point is selected as the initial node, and the polyline grows by searching for neighboring centers within a distance threshold $D_{th}$.

Among these candidates, only those satisfying an angular constraint are considered. Let $v_{prev}$ be the direction vector of the previous segment and $v_{curr}=c_{next}-\;c_{curr}$ be the vector to a candidate point. The candidate is accepted only if the turning angle ϕ satisfies:

$$\phi =arccos\left(\frac{v_{prev}\;\cdot v_{curr}}{\left\|v_{prev}\;\right\|\;\left\|v_{curr}\right\|}\right)<\theta_{th}$$

where $\theta_{th}$ is the predefined angle threshold. Among the valid candidates, the closest point is chosen as the next node, exactly as in Step 27 of Algorithm 1. By iteratively applying this rule, a polyline grows along the direction of the wire harness, as illustrated in Figure 8c.

This procedure is applied repeatedly from multiple starting points, producing a set of candidate polylines. In accordance with Step 34 of Algorithm 1, the polyline containing the largest number of connected OBB centers—that is, the longest valid path—is selected as the main centerline, while very short or fragmented polylines are discarded as invalid. Finally, the selected centerline is smoothed using B-spline fitting, as shown in Figure 8d.

**Algorithm 1** OBB-based Centerline Estimation**Input**:Wire harness point cloud P, Thresholds $N_{min},\;L_{max},\;D_{th},\;\theta_{th}\;\;$
**Output**: Smooth centerline curve $C_{final}$
1:
**//Step 1: Pre-processing (Noise Removal)**
2:      Build graph G from P using connectivity3:      $P^{\prime }$← Largest Connected Component (G)4://**Step 2: OBB-tree Construction**5:      
$Q\;\leftarrow \left\{P^{\prime }\right\};\;$
Centers ← ∅
6:      **while** Q is not empty do7:            
S ←Q.dequeue()
8:            Compute covariance matrix Σ of S
9:            Calculate eigenvectors $e_{1},\;e_{2},\;e_{3}$ and eigenvalues $\lambda_{1},\;\lambda_{2},\;\lambda_{3}$
10:            Construct OBB enclosing S oriented along $e_{1}$
11:            **if** $\left|S\right|<\;N_{min}\;or\;OBB.length<\;\;L_{max}$
            **then**12:                  Centers. add (OBB.center)13:            
**else**14:                  Split S into $S_{1},\;S_{2}$ along plane through mean perpendicular to $e_{1}$
15:                  
$Q.enqueue(S_{1});\;Q.enqueue(S_{2})$
16:            **end if**17:            **end while**18://**Step 3: Polyline Growing**19:      
Polylines←∅
20:      **for each** point $c_{i}\in Centers\;$**as start node do**21:            
$L\leftarrow \;\left\{c_{i}\right\};\;v_{prev}\leftarrow tangent\;of\;OBB\;at\;c_{i}$
22:            **repeat**23:                  Find neighbors $N_{i}=\;\left\{c_{j}\;\right|\;\left\|c_{j}-c_{curr}\right\|<\;D_{th}\}$
24:                  Filter $N_{i}$ by angle: $\left(\frac{v_{prev}\;\cdot (c_{j}-c_{curr})}{\left\|v_{prev}\;\right\|\;\left\|c_{j}-c_{curr}\right\|}\right)<\theta_{th}\;$
25:                  
$c_{best}\leftarrow \arg{min}_{c\in N_{i}}\left\|c-c_{curr}\right\|$
26:                  **if** $c_{best}$ exists **then** $L.append\left(c_{best}\right);Update\;c_{curr},\;v_{prev}\;\;$27:            **else** break 28:            **until** termination29:         
Polylines.add(L)
30:      **end for**31:

$C_{raw}\leftarrow \;LongestPath(Polylines)$

32:

$C_{final}\leftarrow BSplineFit(C_{raw})$

33:return
$C_{\mathrm{final}}$


### 5.2. Generation of Generalized Cylinders

To generate a generalized cylinder, the radius of the wire harness must be estimated at multiple locations along the centerline. For this purpose, cross-section points are computed from the wire-harness point cloud. Because wire-harness point clouds are often sparse and contain missing regions, they are first converted into wireframe models to extract cross-section points more reliably.

It is well known in terrestrial laser scanning that a point cloud acquired from a single scanner position can be mapped onto a two-dimensional grid whose axes correspond to the altitude and azimuth angles of the laser beams [3,4,5]. Each scan position therefore provides a structured 2D grid in angle space, and by connecting adjacent grid points, a wireframe representation can be constructed.

**Figure 9:**
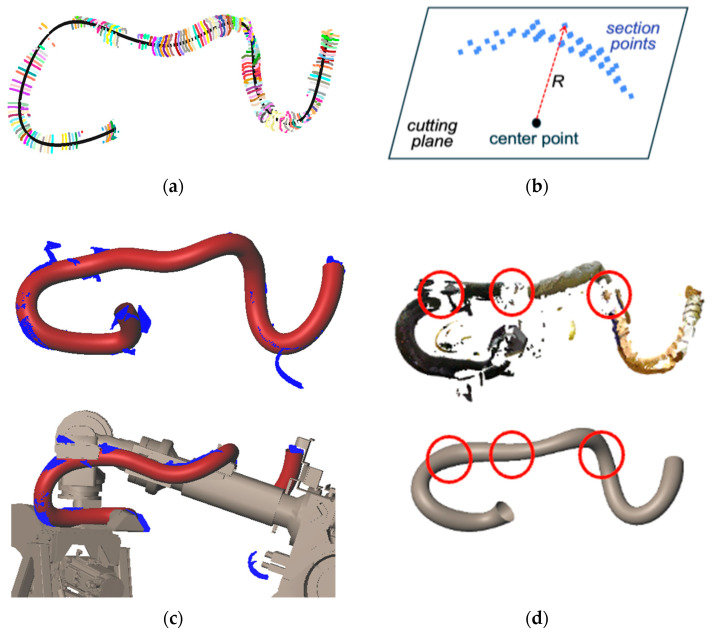
Generation of generalized cylinders for dynamic wire harnesses: (**a**) Cross-section points along the centerline. Different colors indicate different cross-sections.; (**b**) Radius from the farthest section point; (**c**) Generalized cylinder for a wire harness; (**d**) 3D model with interpolated missing points. The red regions indicate the interpolated parts.

Cross-section points are obtained by slicing the wireframe model with cutting planes. These cutting planes are placed at equally spaced intervals along the centerline, and their normals are defined by the tangent directions of the B-spline curve representing the centerline. The intersection between each cutting plane and the wireframe model yields the cross-section points, as illustrated in Figure 9a, where different colors indicate individual cross-sections.

The center of the generalized cylinder is then refined using these cross-section points. When many points are missing, the centers of the OBBs used earlier may not coincide with the true center of the wire harness. Therefore, a circle is fitted to each cross-section using the RANSAC method, and the circle center is adopted as the section center. By computing these centers for all cutting planes, the centerline of the wire harness is refined. The radius at each section is estimated as the distance from the center to the farthest cross-section point to ensure conservative collision avoidance, as shown in Figure 9b.

Local distortions in the wire-harness surface may cause fluctuations in the estimated radii. To obtain a gradual and realistic variation, the radii are treated as a function of the centerline arc length, and a smooth B-spline curve is fitted to this data. The fitted curve is then used to correct the radius values along the centerline.

Finally, the generalized cylinder is generated by interpolating the refined centerline and the corresponding smoothed radius values. The resulting 3D model is shown in Figure 9c presents examples of generated wire-harness models displayed together with an articulated robot.

## 6. Three-Dimensional Model Generation for Branched Wire Harnesses

**Figure 10:**
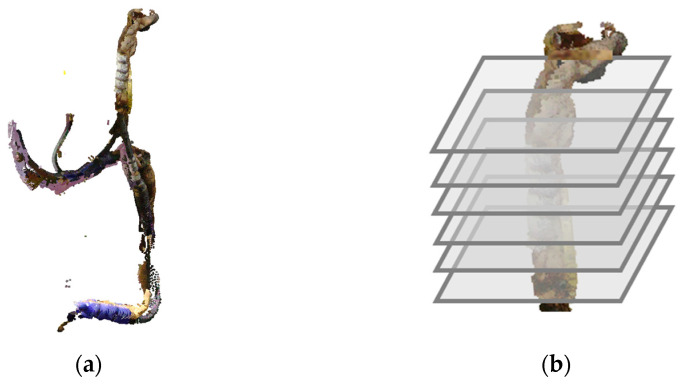

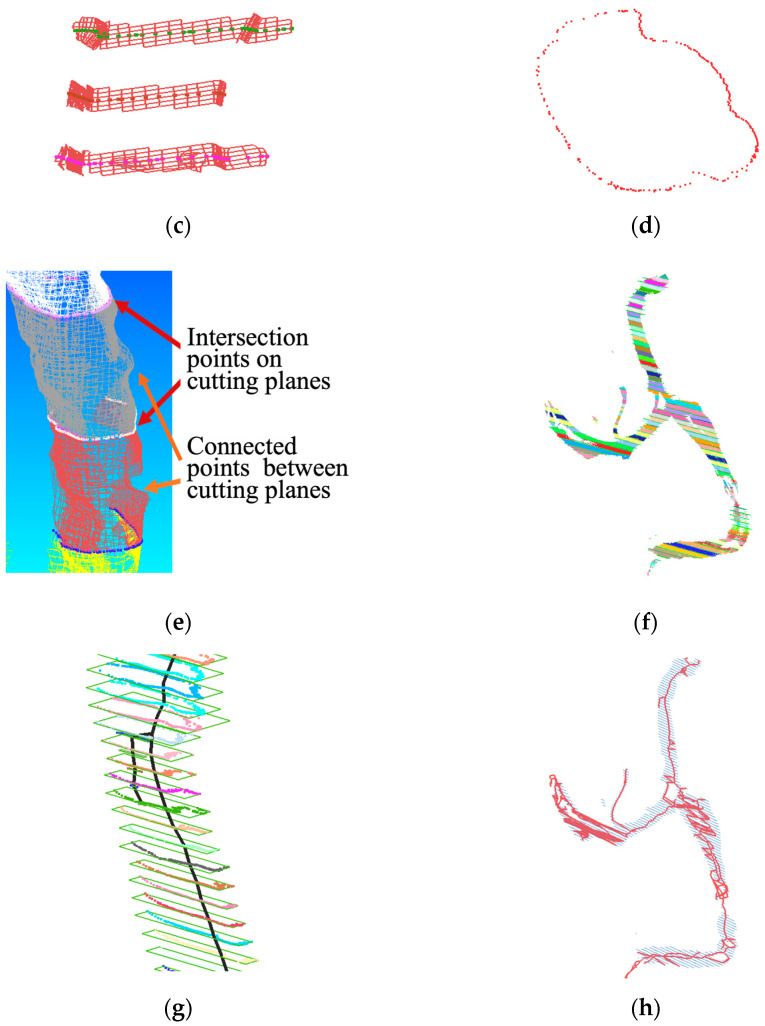
Reeb graph construction process: (**a**) Branched wire harness; (**b**) Cutting planes; (**c**) 2-neighbor edges; (**d**) Connected section points on a cutting plane; (**e**) Edge segments between cutting planes; (**f**) Segmented wire harness point; (**g**) Links defined between neighboring cross-sections; (**h**) Reeb graph.

This section describes the process for estimating the centerline and generating a 3D model for branched wire harnesses. Figure 10a shows an example of a branched wire harness, which contains four endpoints and three branch points. To model such branching structures, we employ a Reeb graph. A Reeb graph is a topological representation that captures the connectivity of a shape and is well suited for expressing the branching structure of wire harnesses.

### 6.1. Reeb Graph Generation

Our method constructs a Reeb graph based on the connectivity of cross-sections extracted from the point cloud. Similarly to the procedure described in the previous section, the point cloud is first converted into a wireframe model. Cutting planes are then generated by applying principal component analysis (PCA) to the point cloud and placing planes at equal intervals along the direction of the first principal component, as illustrated in Figure 10b. The intersections between these cutting planes and the wireframe model provide the cross-section points. At each cutting plane, we retain the wireframe edges that intersect the plane, as well as the resulting intersection points, since the edges carry the connectivity required for detecting connected components.

The cross-section points on each cutting plane are then grouped. As shown in Figure 10c, the 2-neighbors of each intersecting edge are collected, allowing nearby intersection points to be connected through the wireframe edges. As a result, the intersection points belonging to the same cross-section of the wire harness form as a single connected component, as illustrated in Figure 10d.

Next, we track the connectivity of wireframe edges between consecutive cutting planes and divide the edges into segments bounded by the planes. Each segment is then assigned a unique label. Figure 10e illustrates the segments between two cutting planes in different colors, and Figure 10f shows the segmented structure for the entire branched wire harness.

In the Reeb graph, each connected component on a cutting plane is treated as a node. Nodes that share the same labeled edge segment are then connected, as shown in Figure 10g. Finally, the complete Reeb graph is obtained, as illustrated in Figure 10h.

**Figure 11:**
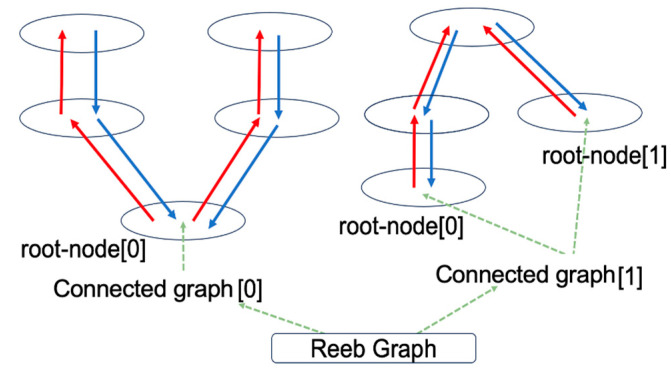
Reeb graph data structure.

In our implementation, this Reeb graph is represented using the bidirectionally linked data structure illustrated in Figure 11. Because the processing pipeline involves multiple steps, such as cutting-plane generation, connected-component detection, and inter-plane connectivity tracking, we summarize the overall logic in Algorithm 2 for clarity. The detailed procedure for constructing the Reeb graph from the point cloud is presented in Algorithm 2.

**Algorithm 2** Reeb Graph Construction for Branched Harnesses
**Input:**
Wire harness point cloud P, Interval $d_{step}\;$
**Output**: Reeb graph $G_{Reeb}=(V,\;E)$
1:
**//Step 1: Initialization**
2:      Construct wireframe W from P by connecting nearest neighbors3:      Compute 1st principal component $v_{pca}$ of P using PCA4:      Generate cutting planes $\Pi_{1},\;\Pi_{2},\;\ldots ,\;\Pi_{N}$ along $v_{pca}$ with interval $d_{step}$
5://**Step 2: Node Generation**6:      V ← ∅
7:      **for** k=1 to N **do**8:            Find intersection points $I_{k}$ between W and $\Pi_{k}$
9:            Group $I_{k}$ into connected components $\left\{C_{k,1},\;C_{k,2},\;\ldots \right\}$ based            on connectivity of edges in W (2-neighbor search)10:            **for each** component $C_{k,j}$
**do**11:                  Create node $v_{k,j}\;\leftarrow$ Centroid $\left(C_{k,j}\right)$

                  Add $v_{k,j}$ to V
12:            **end for**13:      **end for**14://**Step 3: Edge Generation**15:      
E ← ∅
16:      **for** k=1 to N−1
**do**17:            **for each** node $u\;\in \;\Pi_{k}$ and $v\;\in \;\Pi_{k+1}$
**do**18:                  **if** there exists a wireframe edge segment connecting the component of                  u to the component of v
**then**19:                        Add edge (u, v) to E
20:                  **end if**21:            **end for**22:      **end for**
23:**return** $G_{Reeb}=(V,\;E)$

### 6.2. Centerline Detection and Shape Reconstruction

**Figure 12:**
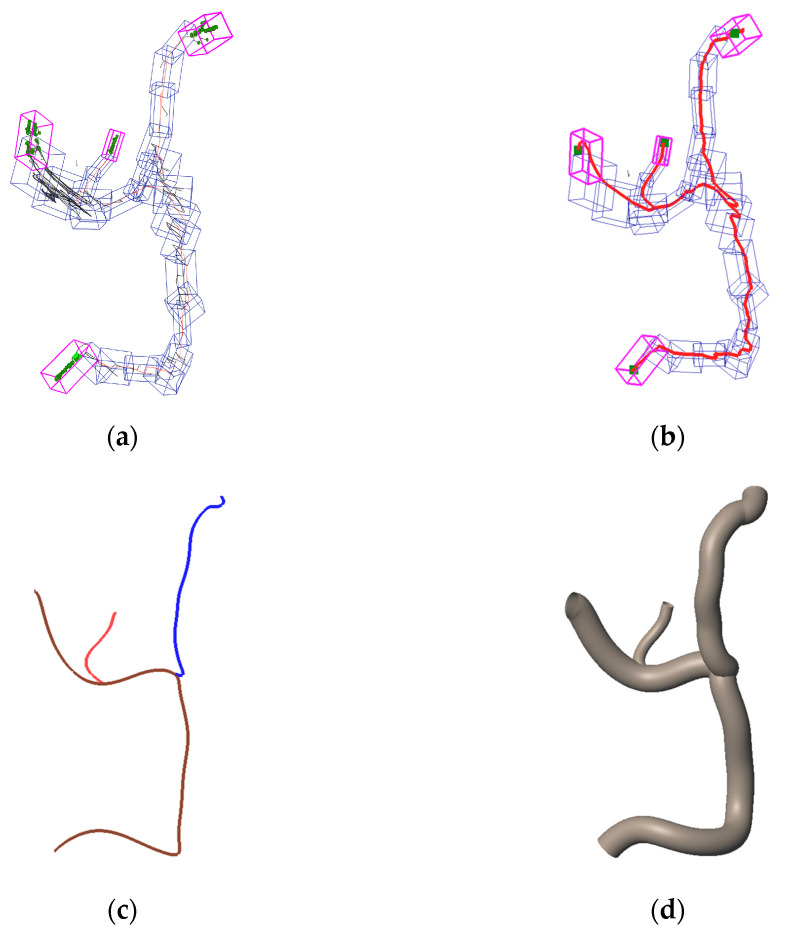
Centerline detection and reconstruction for a branched wire harness: (**a**) OBB-tree of Reeb graph; (**b**) Estimated centerline; (**c**) Smoothed branched centerline; (**d**) 3D mesh model along the centerline.

The Reeb graph shown in Figure 10h captures the global branching structure of the wire harness. However, because it is constructed directly from segmented cross-sections, the resulting graph often contains local noise and many small spurious branches. To obtain a centerline that reflects the global topology rather than these local irregularities, we organize the Reeb graph nodes into spatially coherent groups using an OBB-tree. For each group of nodes, the orientation of its OBB is determined from the spatial distribution of the nodes, while the box dimensions are set large enough to enclose the segment edges associated with those nodes. This produces an OBB-tree that compactly represents the global arrangement of the Reeb graph. Figure 12a shows the resulting OBBs.

To determine which OBBs are adjacent, we evaluate pairwise intersections between boxes. Several methods can be used for this purpose. In our implementation, we use intersection testing based on the Separating Axis Theorem (SAT) [18], which provides an efficient and widely adopted way to check overlap between convex bounding boxes. For each pair of OBBs, 15 separating axes are tested, and if no separating axis is found, the boxes are treated as connected. An endpoint box is defined as an OBB that connects to another box on one side but has no connection on the opposite side. In Figure 12a, endpoint boxes are shown in magenta.

Points contained in these magenta boxes serve as candidate endpoints of the wire harness. To identify a single endpoint from each box, we apply Dijkstra’s algorithm using edge lengths as weights. The shortest-path distances are computed for all pairs of candidate endpoints. Because a branched wire harness contains more than two endpoints, multiple endpoint pairs exist, and therefore multiple shortest paths are obtained. These paths are merged to form a branched centerline, as illustrated in Figure 12b, where the selected endpoints are shown in green.

The resulting polyline centerlines are then smoothed using B-spline fitting to obtain a continuous branched centerline representation, as shown in Figure 12c. Finally, the radius along this centerline is estimated from the point cloud using the method described in Section 5.2, and a complete 3D mesh model is reconstructed, as shown in Figure 12d.

We note that the proposed method assumes that the wire harness does not form a closed-loop structure. This assumption is consistent with our observations in actual manufacturing environments, where dynamic wire harnesses attached to articulated robots are bundled and constrained at their terminal ends by clamps, resulting in open-chain geometries rather than closed loops.

## 7. Experimental Results

### 7.1. Extraction and Segmentation Results

**Figure 13:**
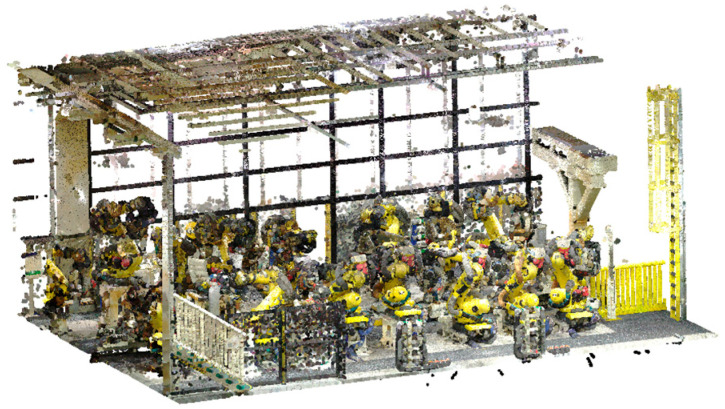
Factory point cloud.

We evaluated the proposed method on six articulated robots installed in different positions and configurations within an automobile manufacturing plant. Figure 13 shows the point cloud of the entire facility, acquired from multiple TLS scanning positions.

**Figure 14:**
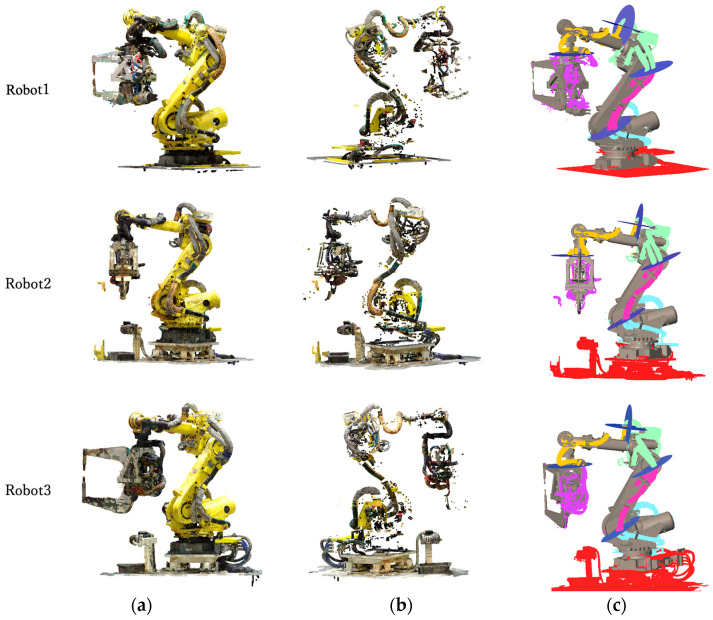
Experimental results s: (**a**) Input point clouds; (**b**) Remaining points after removing the robot links; (**c**) Detected wire harness points for each robot link.

From this dataset, the robot-only point cloud was extracted using the method in [17], as shown in Figure 14a. This extracted robot point cloud served as the input for the subsequent validation. Figure 14b shows the wire-harness candidate points obtained by removing points in the vicinity of the robot links, using the robot CAD models aligned by the method in [17]. Most points corresponding to the robot body were eliminated, although some residual points remained.

Figure 14c presents the result of segmenting the extracted wire-harness point cloud using the defined cutting planes. The harnesses are successfully separated at appropriate boundaries, consistent with their motion characteristics. Based on our visual inspection, the segmented regions correspond well to the intended link-wise divisions of the harnesses. In addition, multiple robot-teaching experts independently reviewed the segmentation results and confirmed that the obtained boundaries are appropriate from the standpoint of practical robot operation. To further validate the segmentation accuracy quantitatively, we manually created ground truth data for two of the six robots. We calculated the Precision, Recall, and F1-score for the segmentation results by comparing the automated classification against the manual labels. The evaluation results are summarized in Table 1. The method achieved high scores across all metrics, confirming that the proposed automated segmentation aligns closely with the ground truth and performs effectively on real-world data.

### 7.2. Computational Efficiency

Next, we evaluated the computation time required to obtain the segmentation results shown in Figure 14c from the candidate points in Figure 14b. In our implementation, this processing is performed completely automatically. The procedure consists of assigning each point to its nearest robot link, removing points that do not belong to any harness, and refining the boundaries using the cutting planes.

Table 2 summarizes the average number of wire-harness candidate points and the average CPU time measured across the six robots. All computations were carried out on a PC equipped with 64 GB of RAM and an Intel Core i9-11900K running at 3.50 GHz. The results show that the wire-harness point clouds can be segmented for each robot link within a practical computation time.

### 7.3. 3D Reconstruction Accuracy

**Figure 15:**
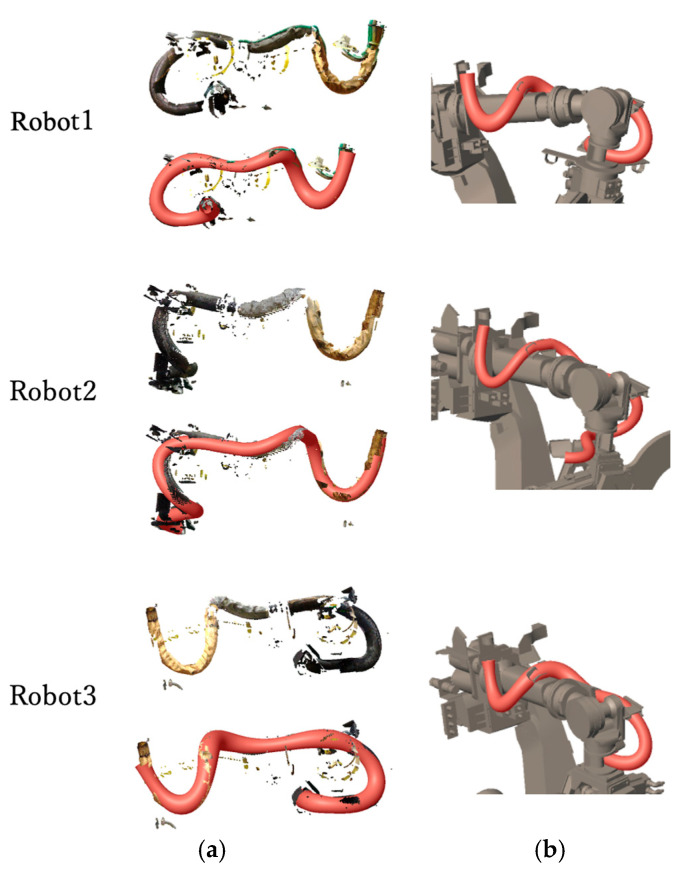
(**a**) 3D model generation results for dynamic wires. (**b**) Overlaying robot links and 3D model of dynamic wire.

Figure 15 shows the shape reconstruction results applied to the point clouds identified as dynamic wire harnesses in Figure 14c. Although the input point cloud in Figure 15a contains missing data and noise, the proposed method reconstructs an appropriate 3D model by smoothly interpolating the missing portions. Figure 15b presents the generated 3D model integrated with the robot’s CAD model.

To evaluate the validity of the estimated harness thickness, we compared the radius of the generated model with that of the measured point cloud. Table 3 summarizes the absolute difference and relative error. The results show a positive relative error of approximately 5%, indicating that the modeled harness is slightly thicker than the measured points. This tendency is consistent with the safety-first design philosophy of our approach.

In addition to radius evaluation, Table 4 reports the mean and root mean square (RMS) distances between each dynamic wire point cloud and its corresponding reconstructed model. For each point in the harness point cloud, the shortest distance to the surface of the reconstructed generalized cylinder was computed. Our radius definition, which uses the farthest cross-section point rather than the average, naturally contributes to the slight overestimation observed in Table 3 but provides a conservative safety margin against collision.

We note that in industrial robot simulation environments, safety clearances on the order of 30–50 mm are typically enforced. The reconstruction errors reported here are significantly smaller than those margins, demonstrating that the proposed method achieves sufficient geometric accuracy for practical interference checking and motion-planning applications.

### 7.4. Discussion

In our implementation, several threshold parameters were used during segmentation and centerline estimation. For the experiments in this section, the threshold values summarized in Table 5 were adopted. These values were determined empirically based on the typical diameter (approximately 30–100 mm) and routing characteristics of industrial wire harnesses. In particular, the OBB length threshold of 10 cm corresponds to the scale of a single local cross-sectional segment of the harness, ensuring that each OBB captures a meaningful geometric portion without merging multiple bends. Similarly, the minimum requirement of 128 points per OBB reflects the density of our TLS scans, providing sufficient samples for stable eigenvector estimation during PCA-based orientation extraction.

These parameter choices introduce certain trade-offs. For the OBB subdivision size, smaller boxes can capture fine local curvature but become more sensitive to residual noise, whereas larger boxes suppress noise but may smooth out sharp geometric transitions. Likewise, for the polyline connection angle, a stricter angular constraint reduces the risk of incorrect branching but may prematurely terminate the growing polyline at sharply curved regions, while a looser constraint allows the curve to follow tighter bends but increases the possibility of connecting to unrelated nearby points.

However, we experimentally confirmed that variations of approximately ±10% in these thresholds did not result in topological errors or significant deviations in the reconstructed geometry. This suggests that as long as the parameters are selected within a range consistent with the target object’s scale, extremely precise fine-tuning is not strictly required. Overall, these observations indicate that the proposed method is not overly sensitive to parameter choices, and that reliable reconstruction can be achieved with relatively coarse parameter tuning.

While the primary focus of this study is geometric reconstruction, the generated 3D models are designed to be directly applicable to dynamic simulation for motion planning. By importing the reconstructed meshes into a physics engine, the harness can be modeled as a deformable linear object. The segmentation results provided by our method allow for the definition of fixed constraints at the clamp positions (attachment points) on the robot links. This setup enables the simulation of realistic harness deformation and collision detection during robot motion, thereby bridging the gap between static reconstruction and dynamic behavioral analysis.

## 8. Conclusions

In this paper, we present a comprehensive method for extracting, segmenting, and reconstructing 3D models of wire harnesses installed on articulated robots. Candidate wire-harness point clouds are first identified by removing the robot geometry through comparison with its CAD model. The extracted points are then classified into static harnesses, which move together with the robot links, and dynamic harnesses, which move independently.

For 3D reconstruction, two complementary centerline estimation strategies were introduced, depending on whether the harness contains branches. An OBB-tree-based approach is applied to unbranched harnesses and remains robust even when the point cloud contains significant occlusion. For branched harnesses, a Reeb-graph-based method was developed to capture the global branching topology. The experimental results on six industrial robots demonstrated that accurate 3D models can be reconstructed from point clouds with mean errors on the order of only a few millimeters and within practical computation times.

There remain several opportunities for further improvement. First, thin-diameter harnesses were difficult to identify with the current approach due to limitations in TLS resolution. In addition, the present method primarily assumes tree-like structures without cycles; future work will extend the framework to handle looped configurations and more complex sagging behaviors by incorporating more advanced topological analysis.

Second, discrepancies between CAD models and actual robot geometry, including unmodeled accessories, may affect extraction accuracy. To address this issue, we plan to investigate learning-based and template-matching approaches for automatically detecting attachment elements such as clamps during the extraction stage. Furthermore, to improve practical usability, we aim to increase the level of automation in selecting adaptive parameters by developing algorithms that optimize parameter values based on local point-cloud characteristics and geometric context.

Finally, we intend to apply the reconstructed 3D models to robot motion-planning and collision-avoidance simulations in operational automotive assembly environments, and to evaluate the scalability of the proposed solution across a wider variety of robotic workcells.

## Figures and Tables

**Table 1:** Segmentation performance metrics compared with ground truth data.

	Robot A	Robot B	Average
Precision	0.951	0.991	0.971
Recall	0.913	0.990	0.952
F1-score	0.923	0.990	0.957

**Table 2:** Average calculation time for segmentation.

Average number of harness points	3.2 million
Average CPU time	34.7 s

**Table 3:** Evaluation of radius estimation: comparison of absolute difference and relative error between measured points and generated models.

	Robot A	Robot B
Measured Radius [mm]	33.2	33.2
Model Radius [mm]	31.6	29.9
Absolute Difference [mm]	1.83	3.31
Relative Error [%]	5.41	9.93

**Table 4:** Mean distance and RMS error between dynamic wire point clouds and reconstructed models.

	Mean Distance [mm]	RMS [mm]
Robot A	8.57	11.7
Robot B	7.70	9.65
Robot C	7.28	8.89
Robot D	7.80	9.48
Robot E	10.6	17.6
Robot F	7.91	9.93
Average	8.31	11.2

**Table 5:** Threshold parameters used in the experiments.

Generating OBB	
Maximum edge length	10 cm
Minimum number of partitions	2
Maximum number of partitions	6
Minimum number of points in the box	128
Connecting centerline	
Cutting plane interval	5 mm
Connection angle	100 degrees
Neighborhood search distance	20 cm
Minimum centerline length for removal	30 cm

## Data Availability

The original contributions presented in this study are included in the article. Further inquiries can be directed to the corresponding author.
